# Examining the association between cognitive ability and emotional problems across childhood using a genetically informative design: could there be a causal relationship?

**DOI:** 10.1111/jcpp.70008

**Published:** 2025-07-16

**Authors:** Meredith X. Han, Ivan Voronin, Margherita Malanchini, Tom A. McAdams

**Affiliations:** ^1^ Social, Genetic and Developmental Psychiatry Centre Institute of Psychiatry, Psychology and Neuroscience, King's College London London UK; ^2^ Yong Loo Lin School of Medicine National University of Singapore Singapore Singapore; ^3^ Université Laval Quebec City QC Canada; ^4^ School of Biological and Behavioural Sciences Queen Mary University of London London UK

**Keywords:** Cognition, internalising disorder, behavioural genetics, longitudinal studies

## Abstract

**Background:**

Emotional problems co‐occur with difficulties in verbal and nonverbal cognitive ability, yet the pathways underlying their association remain poorly understood: It is unclear whether effects may be causal, and to what extent they may run from cognition to emotion, or vice versa.

**Methods:**

Our preregistered analyses included 5,124 twin pairs from the Twins Early Development Study (TEDS). At ages 7, 9 and 12, emotional problems were assessed through the strengths and difficulties questionnaire, and cognition was assessed using task‐based measures. Cross‐lagged models examined the influence of cognition and subdomains of verbal and nonverbal abilities on emotional problems and vice versa, across development. Genetic cross‐lagged models examined the effect of cognition on emotional problems and vice versa, after controlling for shared genetic and environmental influence.

**Results:**

Cross‐lagged paths in both directions were observed between cognitive ability and emotional problems (from −0.11 to −0.05). Cross‐lagged associations that persisted after accounting for common genetic and environmental influences were between nonverbal ability and emotional problems. Higher emotional problems at age 7 predicted lower nonverbal ability at age 9, with 22% of the phenotypic association remaining. This, in turn, predicted greater emotional problems at age 12, with 13% of the association remaining.

**Conclusions:**

Genetic and environmental factors accounted for a large proportion of the cross‐lagged associations. Emotional problems in early childhood could result in a cascade effect, leading to lower nonverbal cognition in middle childhood, which increases the risk of emotional problems in late childhood. These findings highlight the importance of age‐ and domain‐specific interventions.

## Introduction

Individual differences in cognitive performance are associated with mood disorders. Children with major depressive disorder display deficits in memory, verbal fluency, sustained attention and inhibitory control (Wagner, Muller, Helmreich, Huss, & Tadic, 2015). Similarly, outside of the clinical population, verbal and nonverbal cognitive deficits are observed in children with elevated emotional and behavioural issues (Bornstein, Hahn, & Suwalsky, 2013; Plomin, Price, Eley, Dale, & Stevenson, 2002; Salmon, O'Kearney, Reese, & Fortune, 2016). Cognitive ability may also have long‐lasting effects on well‐being, such that those with higher childhood cognitive performance have lower odds of recurrent depressive episodes and suicidal ideation in adulthood (Hung et al., 2016).

Cognitive ability is an umbrella term capturing common variance from fluid reasoning, visual and auditory perception, and processing speed (Carroll, 1993), higher order executive function domains (i.e. working memory) (Diamond, 2013; Engelhardt et al., 2016) and crystallised ability (i.e. acquired knowledge) (Cattell, 1963). These skills group into verbal and nonverbal cognitive abilities (Wechsler, 1992). Verbal ability refers to the use of language, while nonverbal ability refers to working memory, perceptual reasoning and processing speed. The cognitive reserve theory of psychopathology suggests that cognitive ability protects against emotional problems by helping children navigate developmental challenges such as anxieties in educational settings (Masten & Curtis, 2000; Venezia et al., 2018). Conversely, emotional problems may impede learning due to distractions or poor school attendance, delaying cognitive development (Ford et al., 2018). Different skillsets may be more important at various stages of development, with nonverbal ability playing a larger role in late childhood for academic and social competence (Masten & Curtis, 2000; Morgan, Farkas, Hillemeier, Hammer, & Maczuga, 2015; Nelson, Welsh, Trup, & Greenberg, 2011; Salmon et al., 2016). Verbal ability enables children to engage in conversations and internalise rules and guidelines, supporting the development of self‐regulation skills (Salmon et al., 2016). Evidence suggests that verbal ability may be more strongly linked to emotional difficulties in early childhood, but this association may weaken as children grow older due to the development of effective compensatory strategies (Beitchman, Brownlie, & Bao, 2014).

Despite consistent correlational evidence suggesting an association between cognitive ability and emotional problems, limited studies examine the separate influence of verbal and nonverbal abilities (Donati, Meaburn, & Dumontheil, 2021; Flouri et al., 2019; Papachristou & Flouri, 2020). Studies find bidirectional associations between language ability and emotional problems across childhood (Helland, Roysamb, Wang, & Gustavson, 2018; Tamayo et al., 2024). For nonverbal ability, findings are inconsistent on the direction of associations. One study shows that internalising problems at age 10 predict working memory deficits at age 14, with no associations from age 10 working memory deficits to age 14 internalising (Donati et al., 2021). In younger children, nonverbal cognitive deficits precede internalising problems (Patwardhan, Nelson, McClelland, & Mason, 2021) or associations are bidirectional (Romer & Pizzagalli, 2021). It remains unclear if one ability is more strongly associated with emotional problems than the other, and if the strength of these associations varies across development (Plomin et al., 2002; Weeks et al., 2014). Furthermore, it is yet to be established whether the association between emotional disorders is attributable to common causes and/or may have a causal component. Common genetic causes and family factors such as social and economic disadvantages explain a portion of the correlation between psychiatric disorders and general cognitive ability (Elsayed, Luby, & Barch, 2023; Griffith, Crawford, Oppenheimer, Young, & Hankin, 2019; Grotzinger, Cheung, Patterson, Harden, & Tucker‐Drob, 2019; von Stumm, Malanchini, & Fisher, 2023).

Data on twin pairs can be used to evaluate quasi‐causal hypotheses by testing whether associations between variables remain (and thus may be causal) after accounting for shared genetic effects and environmental effects on traits, or whether they do not (and thus are likely not causal) (De Moor, Boomsma, Stubbe, Willemsen, & De Geus, 2008; Duffy & Martin, 1994; Heath et al., 1993; Kohler, Behrman, & Schnittker, 2011; McAdams et al., 2020; Turkheimer & Harden, 2014). Twin models compare resemblance within identical (monozygotic; MZ) twin pairs, who share 100% of their genetic variation and nonidentical (dizygotic; DZ) twin pairs, who share 50% of their genes, to estimate the role of genetic (A), shared (C) and nonshared (E) environmental factors on trait variance. Multivariate twin models are based on cross‐twin correlations for two or more traits. A greater correlation in MZ than DZ twin pairs indicates an additive genetic correlation between the two traits (rA), while an absence of this difference would indicate an environmental correlation (rC). Significant nonshared environment (rE) paths between traits indicate that the associations remain after controlling for influences shared by twins.

To date, twin models have not been used to explore whether the association between cognition and mood disorders may be causal, nor to distinguish the direction of effects. However, twin data has been used to assess the underlying aetiological overlap. Researchers examined associations between emotional problems and verbal and nonverbal cognitive ability at ages 2–4 (Plomin et al., 2002). Results showed stronger genetic and environmental correlations between general psychopathology and non‐verbal than verbal cognitive ability. Shared environmental correlation (rC) explained more than half of the association while genetic effects (rA) explained a third of the association and nonshared environment (rE) explained less than a third, suggesting that there may be small causal effects remaining between nonverbal ability and general psychopathology in preschool years. The potential for causal associations between cognitive ability and psychopathology also extends into adolescence. Researchers found that nonshared environmental factors capture a small proportion of associations from cognitive ability at age 9 to general psychopathology at ages 12 and 16 (von Stumm et al., 2023).

The present preregistered study examined (1) whether associations differ in strength and direction between verbal and nonverbal abilities and (2) whether associations between cognitive ability (general, verbal and nonverbal abilities) and emotional problems remain after controlling for shared genetics and environmental factors. Using longitudinal data, biometric genetic versions of autoregressive ACE cross‐lagged models were fitted to measures collected at age 7, 9 and 12. These models included cross‐lagged prospective predictions between cognitive ability and emotional problems, testing whether these predictions remained significant accounting for shared genetic and environmental influences common to both variables (Malanchini et al., 2017; McAdams et al., 2020). Bidirectional associations were simultaneously examined. We hypothesised that (a) verbal ability would account for a larger proportion of the association at age 7–9 while nonverbal ability would explain a larger proportion of the associations from 9 to 12 and (b) nonshared environmental contributions to the association between cognitive ability (including verbal and nonverbal domains) and subsequent emotional symptoms would be significant (rE >0).

## Method

### Sample

The Twins Early Development Study (TEDS) is a prospective longitudinal study consisting of families of twins born in England and Wales from 1994 through 1996 across all socioeconomic backgrounds. TEDS is representative of the UK population census data (Lockhart et al., 2023). TEDS project approval (05.Q0706/228) was granted by the ethics committee for the Institute of Psychiatry, Psychology and Neuroscience at King's College London. Parents provided written informed consent prior to data collection, and all children assented to the study at 7 years of age.

### Measures

#### Child emotional problems

Emotional problems were measured at ages 7, 9 and 12 using the five‐item emotional problems subscale in the Strength and Difficulties Questionnaire (SDQ) (Goodman, 1997). This is a parent‐reported questionnaire on negative behaviours displayed by the child over the last 6 months (they have many worries). A higher composite score indicated greater emotional problems. The SDQ is a validated screening tool with a Cronbach's alpha of 0.60–0.68.

#### Age 7 cognitive ability

Verbal and nonverbal abilities were assessed by telephone interviews, using a test booklet mailed to parents containing two verbal tests: a 13‐item similarity test (In what ways are a cat and mouse alike?) and an 18‐item vocabulary test (what is a clock?) from the Wechsler Intelligence Scale for Children (WISC‐III‐UK) (Wechsler, 1992), and two nonverbal tests: a 21‐item WISC picture completion task and a nine‐item conceptual groupings task from the McCarthy Scales of Children's Abilities (McCarthy, 1972; Petrill, Rempell, Oliver, & Plomin, 2002). The nonverbal picture completion was the only timed task where children were given 20 s to provide their answer. If 20 s passed and no answer was provided, it was treated as an incorrect response. Verbal and nonverbal composites were created using the mean of the standardised test scores within each domain. A general cognitive ability score was derived by taking the mean of the two standardised verbal and nonverbal test scores. The general cognitive ability composite from the telephone‐administered battery correlated 0.62 with the in‐person Stanford‐Binet Intelligence Scale (Kovas et al., 2007). Similarly, the vocabulary task correlated 0.70 with teacher assessments of reading in the TEDS sample.

#### Age 9 cognitive ability

Cognitive abilities were assessed with test booklets which were mailed and administered under the supervision of the parent. This contained two verbal tests: a 20‐item vocabulary test (what does ‘migrate’ mean?) and an 18‐item general knowledge (in what direction does the sun set?), adapted from the multiple‐choice version of the WISC‐III‐UK (Wechsler, 1992) and two nonverbal tests: the first being a 24‐item puzzle test adapted from the Cognitive Abilities Test 3 (Davis, Arden, & Plomin, 2008; Smith, Fernandes, & Strand, 2001). This involved a spatial reasoning in which, the child was asked to identify which shape out of five, continues a series of shapes. The second nonverbal task was a 24‐item shapes test that assessed inductive and deductive reasoning (a rectangle and a square relates to each other like an oval and what other shape?). The child was asked to identify the shape out of five choices. Nonverbal tests had a high internal consistency of 0.91 for the Puzzle and 0.85 for the Shapes task. Cronbach's alpha were 0.63 for the Vocabulary and 0.63 for the General Knowledge test.

#### Age 12 cognitive ability

Testing was conducted at home via computers using a web‐based programme. The verbal and two nonverbal tests which were previously used at age 9 were age‐matched and used (Kaplan, Fein, Kramer, Delis, & Morris, 1998; Raven & Court, 1998; Wechsler, 1992). These web‐based tasks show good internal consistency of 0.73 for the Puzzle, 0.75 for the Shapes, 0.88 for vocabulary and 0.81 for general knowledge. All the tasks measured using different methods demonstrated moderate to high convergent validity for verbal (*r* = .30 to .56) and nonverbal (*r* = .24 to .40) (Malanchini et al., 2021).

### Statistical analysis

Twins with severe medical problems currently or at birth and whose mothers reported severe medical problems during pregnancy were excluded from the analysis (*n* = 1,076 children). The final analysis sample included 10,247 twins (5,124 families) for whom general cognitive ability data were available at age 7. Of those, 6,377 twins also contributed data at age 9, and 8,444 twins contributed data at age 12. We controlled for the effects of sex and age by regressing our variables on sex and age at each wave and using the residuals of these regressions in subsequent twin analyses (McGue & Bouchard, 1984).

We used an autoregressive cross‐lagged model to examine the phenotypic relation between cognitive ability (including verbal and nonverbal components) and emotional problems at ages 7, 9 and 12. An autoregressive cross‐lagged model specifies autoregressive paths that link measures of the same construct across time points (i.e. stability paths), paths that connect different measures within the same time point (i.e. correlational paths) and paths that connect different measures across time points (i.e. cross‐lagged paths). The cross‐lagged paths are partial regressions, adjusting for stability and correlational associations between cognitive ability and emotional problems. Three separate models were computed to model relationships between emotional problems and (1) general cognitive ability, (2) verbal ability independent of nonverbal ability and (3) nonverbal ability independent of verbal ability. In model 2, we regressed the effects of no‐verbal ability out of verbal ability, and in model 3, we regressed the effects of verbal ability from nonverbal ability. To account for the nonindependence of observations, we clustered twin pairs as a family unit in the analyses.

**Figure 1:**
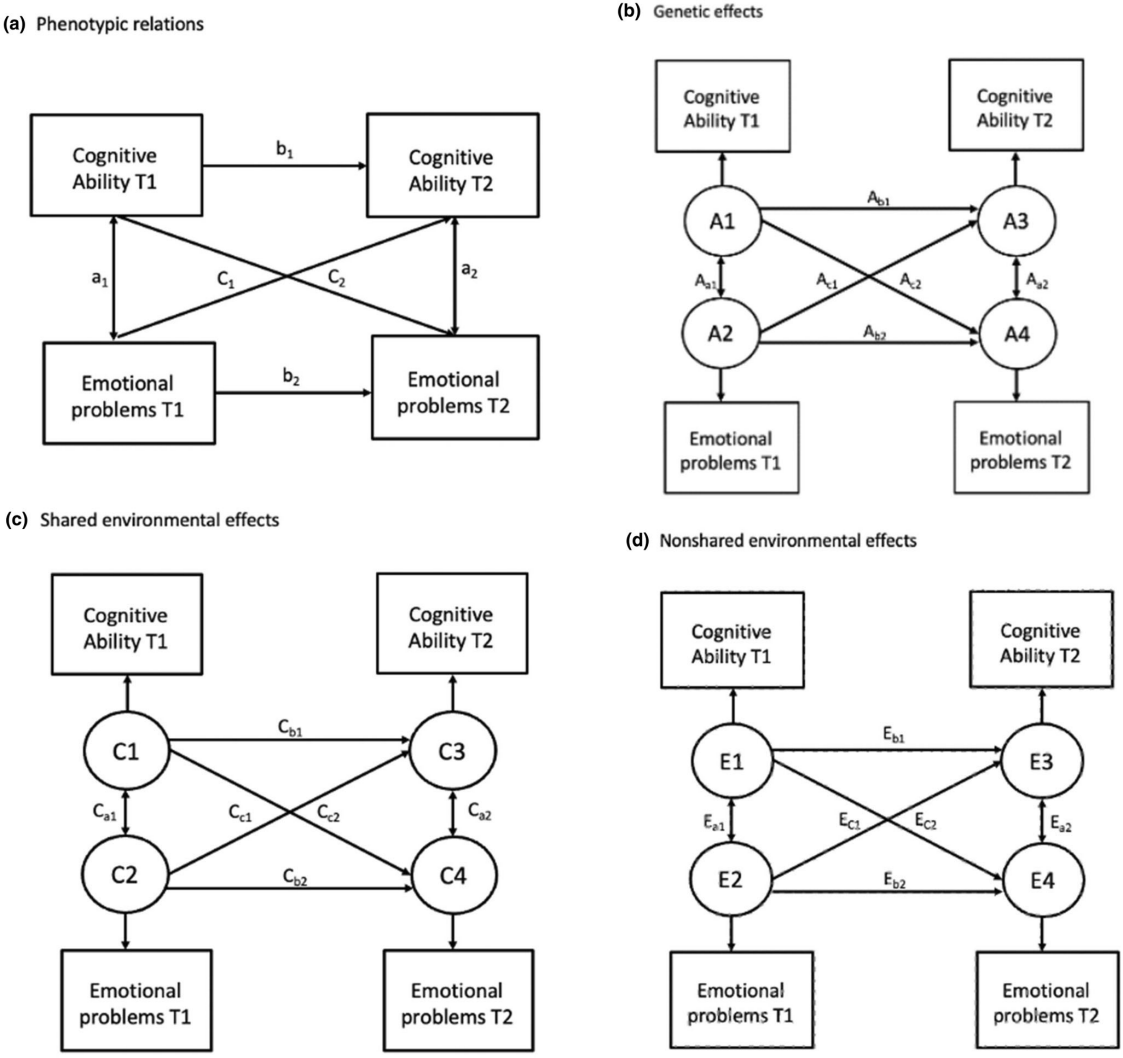
Phenotypic cross‐lagged model (panel a) and ACE cross‐lagged models (panel b–d) for testing the direction of association between cognitive ability and emotional problems. The model examines influences across time‐point 1 (T1) and time‐point 2 (T2). Paths a represent correlational paths between two measures within time, paths b represent associations with the same measure across time and paths c represent cross‐lagged associations between two different measures across time. ACE cross‐lagged models further decompose these paths a–c into genetic (A; panel b), shared environment (C; panel c) and nonshared environment (E; panel d) components. A causal effect of cognitive ability at T1 on emotional problems at T2 can be inferred if, after estimating the variance in emotional problems that can be explained by cognitive ability's A and C components, there should be remaining variance that can also be explained by cognitive ability's E component

Using a structural equation modelling approach, we next fitted autoregressive ACE cross‐lagged models to the data, which decompose the stability, correlational and cross‐lagged paths in to additive genetics, shared environment and nonshared environment components (Figure 1) (Malanchini et al., 2017; Neale & Cardon, 2013). Multivariate twin models, such as the autoregressive ACE cross‐lagged model, are based on cross‐twin cross‐trait correlations. When the estimates of rA, rC and rE are obtained, it is possible to calculate what percentage of the phenotypic (i.e. measured) correlation between two traits is explained by genetic and environmental factors. In the cross‐lagged ACE model (Figure 1), the autoregressive structures of genetic and environmental factors is estimated from the matrix of cross‐twin cross‐trait correlations, and then these estimates are used to compute the relative contribution of A, C and E to each phenotypic path (Malanchini et al., 2017). Evidence for unique environment (E) influence on paths can be interpreted as indicating that a given association remains after controlling for influences shared by twins (McAdams et al., 2020; Turkheimer & Harden, 2014). Using ACE cross‐lagged models as a causal inference method is based on the observation that all traits are influenced, to some extent by E (environmental influences not shared by twins) (Turkheimer & Harden, 2014). Therefore, if X causes Y then the effects of E on X will also explain a proportion of variance in Y (cross‐lagged paths) (Figure 1). However, this comes with the caveat that associations could still be attributable to nonshared confounders, such as an illness that only one twin experiences, that leads to concurrent poor cognitive ability and emotional problems, resulting in a spurious association between the two traits. As such, multivariate twin models cannot *prove* whether an association is causal in nature but an absence of E would serve as evidence against a possible causal effect. An anonymous reviewer of this manuscript asked why we chose not to perform a random intercept cross‐lagged panel model (RI‐CLPM). The RI‐CLPM estimates the effect of increasing the exposure by one unit around the person mean (within‐individual) (Orth, Clark, Donnellan, & Robins, 2021). Specifically, it shows how within‐individual fluctuations in cognitive ability ‘causes’ within‐individual changes in emotional problems, relative to an individual's baseline emotional state. This is a slight variation from our current research question. We chose an autoregressive cross‐lagged panel model as our aim is to examine whether reduced cognitive ability prospectively predicts higher emotional problems *between* individuals. Thus, the cross‐lagged estimates produced in this study reflect the effect of individual differences in trait X on the change in individual differences in trait Y.

The use of twin models to examine causal hypotheses may be unfamiliar to some readers. However, as a quasi‐experimental approach to control for unmeasured confounders, it is well supported by theory and practice (De Moor et al., 2008; Duffy & Martin, 1994; Heath et al., 1993; Kohler et al., 2011; McAdams et al., 2020). In addition to our biometric ACE cross‐lagged model, we also used another twin method that is perhaps more commonly associated with the testing of causal hypotheses, the MZ twin differences design (Supplementary Methods: Appendix S1). However, the MZ differences design reduces the variance by only utilising data from MZ twins. We therefore opted to present the biometric ACE cross‐lagged model as it provides a more robust methodological approach, incorporating data from MZ and DZ twins.

Analyses were conducted in R (R Core Team, 2024) with packages: OpenMx (Neale et al., 2016), TwinAnalysis (https://github.com/IvanVoronin/TwinAnalysis) and Lavaan (Rosseel, 2012) using full information maximum likelihood (FIML). Initially, we ran our models with all paths freely estimated. Where this model did not converge, we ran a correlated factors twin model to identify obsolete (nonsignificant) associations. Based on these results, we removed common environment paths (C) that were nonsignificant from the cross‐lagged models, improving the model's convergence. All analyses performed were preregistered prior to data access: https://osf.io/f4dgq.

## Results

Descriptive statistics for measures of cognitive ability and emotional problems at each age are reported in Table S1 and their intercorrelations in Table S2. Small negative cross‐sectional correlations were found between emotional problems and general cognitive ability (*r* = −.04 to −.16), verbal ability (*r* = −.06 to −.12) and nonverbal ability (*r* = −.01 to −.15). The strongest relationship between general cognitive ability and emotional problems was demonstrated at age 9 (*r* = −.16).

### Phenotypic associations between general cognitive ability and emotional problems

**Figure 2:**
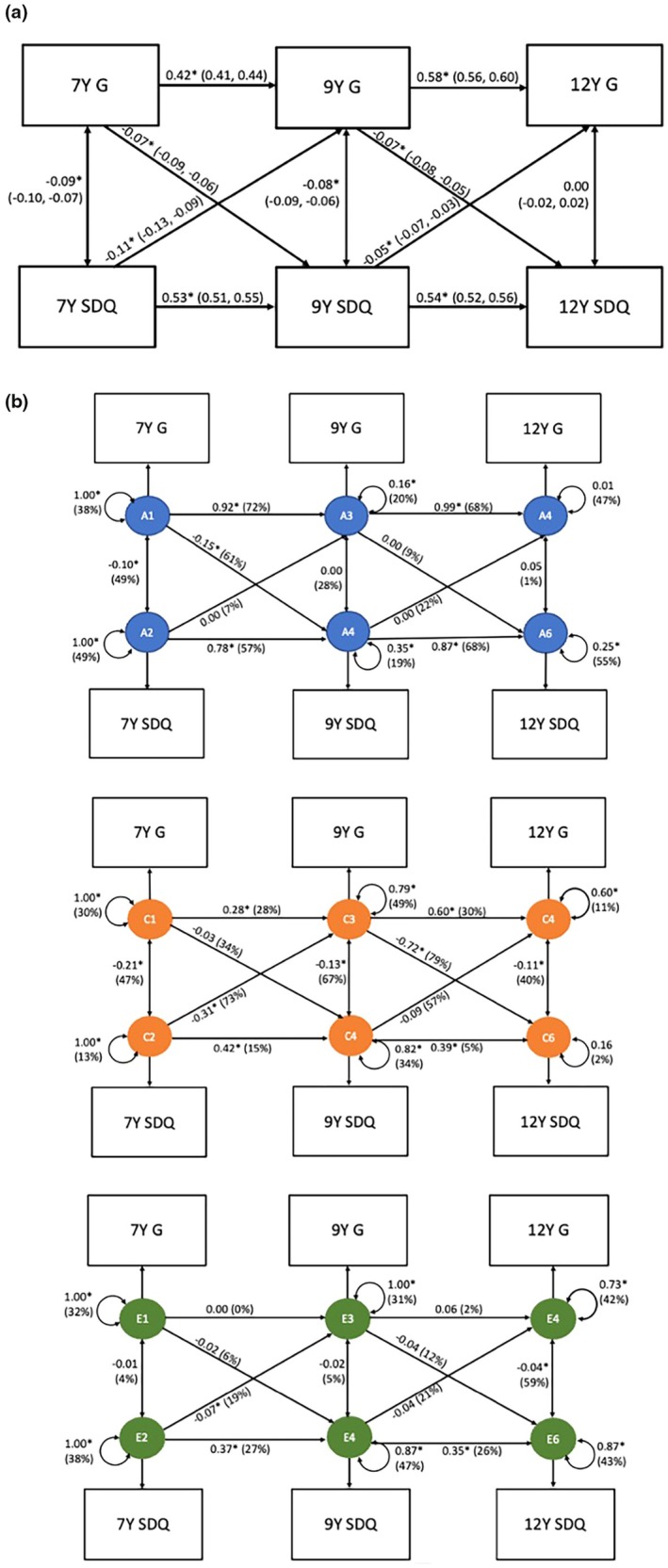
(a) Cross‐lagged model of general cognition and emotional problems at age 7, 9 and 12. (b) Genetic cross‐lagged model of general cognition and emotional problems at age 7, 9 and 12. Standardised beta estimates (95% confidence intervals), G, general cognition, SDQ, Strength and Difficulties Questionnaire: emotional problems subscale. 7Y = age 7, 9Y = age 9, 12Y = age 12. Figure 2a presents phenotypic associations between G and SDQ across time. Figure 2b presents the ACE cross‐lagged model. The three panels (A, C, E) show the standardised beta estimate (% of variance) that is accounted for by genetic effects (A), environmental factors that are shared between twins (C) and environmental factors that are unique to each child (E). Dashed lines indicate the paths that were dropped from the model. The double‐headed arrows are univariate ACE estimates for G and SDQ; specifically, they indicate the ACE decompositions of G and SDQ's phenotypic variance at the first assessment (i.e. age 7 years) and of G and SDQ's respective residual variance at each subsequent assessment (i.e. ages 9 and 12). *p < .05

Figure 2a reports the estimates from the phenotypic cross‐lagged models between general cognitive ability and emotional problems. General cognitive ability and emotional problems were stable across development, where the stability paths ranged from 0.42 to 0.58. Cross‐lagged paths indicate that higher general cognitive ability prospectively predicted lower emotional problems at age 7–9 (β = −0.07, 95% CI: −0.09, −0.06) and 9–12 (β = −0.07, 95% CI: −0.08, −0.05). Similarly, higher emotional problems prospectively predicted lower general cognitive ability at age 7–9 (β = −0.11, 95% CI: −0.13, −0.09) and at age 9–12 (β = −0.05, 95% CI: −0.07, −0.03).

### Could there be a causal effect of general cognitive ability on emotional problems and/or vice versa?

We applied an ACE cross‐lagged model to decompose associations between general cognitive ability and emotional problems into A, C and E components. All models converged and provided good fit to the data compared to a baseline saturated model (Table S8).

Table S3 reports the relative contribution of A, C and E on cognition and emotional problems. The heritability of general cognitive ability (*h* ^2^ = 0.38 at age 7 to *h* ^2^ = 0.47 at age 12) and emotional problems increased across development (*h* ^2^ = 0.49 at age 7 to *h* ^2^ = 0.55 at age 12), in line with previous findings (Haworth et al., 2009; Hoekstra et al., 2007). The relative contribution of shared environment decreased for general cognition (*c* ^2^ = 0.30 at age 7 to *c* ^2^ = 0.11 at age 12) and emotional problems (*c* ^2^ = 0.13 at age 7 to *c* ^2^ = 0.02 at age 12), while the relative contribution of unique environment on general cognition (*e* ^2^ = 0.32 at age 7 to *e* ^2^ = 0.42 at age 12) and depression (*e* ^2^ = 0.38 at age 7 to *e* ^2^ = 0.43 at age 12) increased. An exception to this trend is found at age 9 (*c* ^2^ = 0.34 for emotional problems and *c* ^2^ = 0.49 for general cognition).

Figure 2b illustrates the ACE cross‐lagged model for general cognitive ability. Table S4 presents the standardised beta estimates and their 95% confidence intervals. Although phenotypic observations showed cross‐sectional associations between general cognitive ability and emotional problems across time, after accounting for shared genetic and environmental influences, the model only yielded one significant cross‐lagged E estimate. A unique environmental contribution was only found for the path from age 7 emotional problems to age 9 general cognitive ability (*E* = 19%), suggesting that high emotional problems at age 7 may cause lower cognitive ability at age 9.

Three nonsignificant E cross‐lagged paths were found: the cross‐lagged paths from general cognitive ability at age 7 to emotional problems at age 9, age 9 cognitive ability to age 12 emotional problems and age 9 emotional problems to age 12 cognitive ability. Age 7 general cognitive ability to age 9 emotional problems was mostly accounted for by genetic factors (*A* = 61%). Common environmental factors accounted for a large proportion of variance in paths from age 9 cognitive ability to age 12 emotional problems (*C* = 79%), and in the reverse direction: age 7 emotional problems to age 9 cognition (*C* = 73%). A, C, E estimates for the path from age 9 emotional problems to age 12 cognitive ability were not significant, indicating that we did not have power to decompose due to the small phenotypic association (−0.05).

### Phenotypic associations between verbal performance and emotional problems

We examined associations between subdomains of cognitive ability and emotional problems by assessing the unique contribution of verbal and nonverbal abilities after accounting for the variance explained by each other.

**Figure 3:**
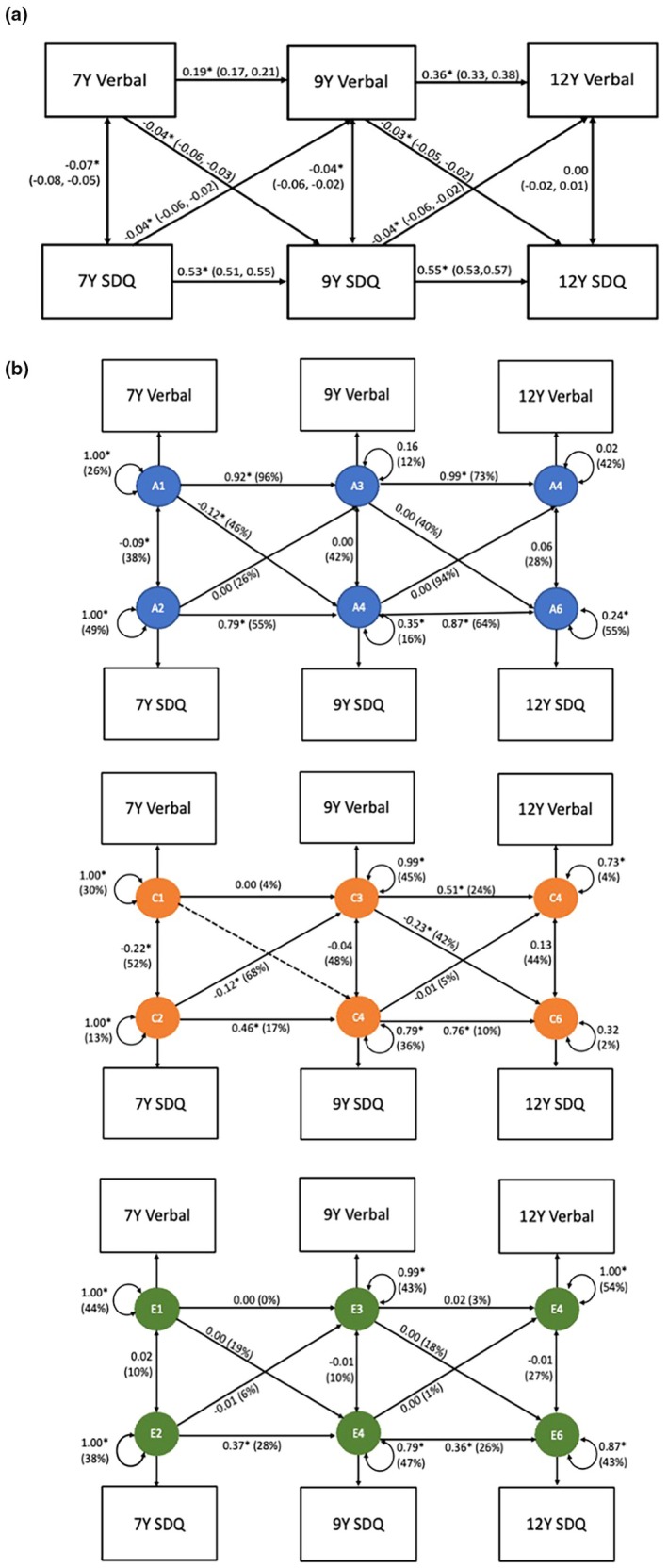
(a) Cross‐lagged model of verbal ability and emotional problems at age 7, 9 and 12 (b) Genetic cross‐lagged model of verbal ability and emotional problems at age 7, 9 and 12. Standardised beta estimates (95% confidence intervals), SDQ, Strength and Difficulties Questionnaire: emotional problems subscale. 7Y = age 7, 9Y = age 9, 12Y = age 12. Figure 3a presents phenotypic associations between verbal performance and SDQ across time. Figure 3b presents the ACE cross‐lagged model. The three panels (A, C, E) show the standardised beta estimate (% of variance) that is accounted for by genetic effects (A), environmental factors that are shared between twins (C) and environmental factors that are unique to each child (E). Dashed lines indicate the paths that were dropped from the model. The double‐headed arrows are univariate ACE estimates for verbal and SDQ; specifically, they indicate the ACE decompositions of verbal and SDQ's phenotypic variance at the first assessment (i.e. age 7 years) and of verbal and SDQ's respective residual variance at each subsequent assessment (i.e. ages 9 and 12). *p < .05

Figure 3a reports the phenotypic cross‐lagged models for verbal ability and emotional problems. Phenotypic cross‐lagged paths indicate that higher verbal ability prospectively predicted lower emotional problems at age 9 (β = −0.04, 95% CI: −0.06, −0.03) and at age 12 (β = −0.03, 95% CI: −0.05, −0.02). Higher emotional problems prospectively predicted lower verbal ability at age 9 (β = −0.04, 95% CI: −0.06, −0.02) to age 12 (β = −0.04, 95% CI: −0.06, −0.02).

### Could there be a causal effect of verbal performance on emotional problems and vice versa?

The relative contribution of genetic (*h* ^2^ = 0.26 at age 7 to *h* ^2^ = 0.42 at age 12) and unique environmental factors (*e* ^2^ = 0.44 at age 7 to *e* ^2^ = 0.54 at age 12) on verbal performance increased across development. Shared environment influences decreased across development, with the exception of age 9 (*c* ^2^ = 0.45) (Table S3).

Although, phenotypic observations showed cross‐lagged associations between verbal ability and emotional problems across time, after accounting for shared genetic and environmental influences in all paths, no unique environmental associations remained in either direction. This indicates no evidence for a causal effect of verbal performance on emotional problems or vice versa. Instead, the cross‐lagged path running from verbal performance to emotional problems was explained by shared genetics at age 7 (*A* = 46%) and marginally by shared environment at age 9 (*C* = 42%), with large confidence intervals (β = −0.23, 95% CI: −0.91, −0.01) (Figure 3b and Table S5). Similarly, shared environment explained a large proportion of variance in the cross‐lagged path from emotional problems to verbal performance at age 7 (*C* = 68%).

### Phenotypic cross‐lagged associations between nonverbal ability and emotional problems

**Figure 4:**
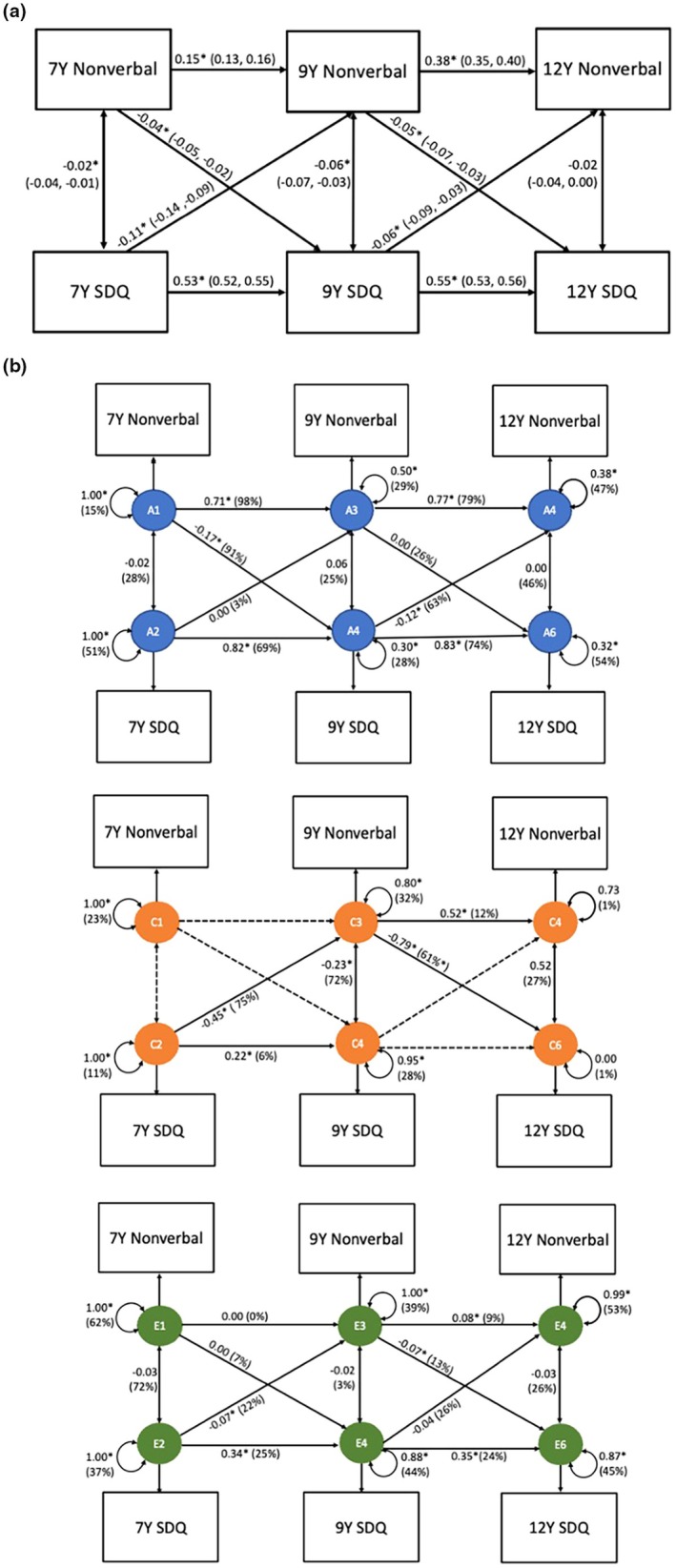
(a) Cross‐lagged model of nonverbal ability and emotional problems at age 7, 9 and 12 (b) Genetic cross‐lagged model of nonverbal ability and emotional problems at age 7, 9 and 12. Standardised beta estimates (95% confidence intervals), SDQ, Strength and Difficulties Questionnaire: emotional problems subscale. 7Y = age 7, 9Y = age 9, 12Y = age 12. Figure 4a presents phenotypic associations between nonverbal performance and SDQ across time. Figure 4b presents the ACE cross‐lagged model. The three panels (A, C, E) show the standardised beta estimate (% of variance) that is accounted for by genetic effects (A), environmental factors that are shared between twins (C) and environmental factors that are unique to each child (E). Dashed lines indicate the paths that were dropped from the model. The double‐headed arrows are univariate ACE estimates for nonverbal and SDQ; specifically, they indicate the ACE decompositions of nonverbal and SDQ's phenotypic variance at the first assessment (i.e. age 7 years) and of nonverbal and SDQ's respective residual variance at each subsequent assessment (i.e. ages 9 and 12). *p < .05

Cross‐lagged paths indicate that higher nonverbal ability prospectively predicted lower emotional problems at age 9 (β = −0.04, 95% CI: −0.05, −0.02) and at age 12 (β = −0.05, 95% CI: −0.07, −0.03) (Figure 4a). Higher emotional problems prospectively predicted nonverbal ability at age 9 (β = −0.11, 95% CI: −0.14, −0.09) and age 12 (β = −0.06, 95% CI: −0.09, −0.03).

### Could there be a causal effect of nonverbal performance on emotional problems and vice versa?

The relative contribution of genetic effects (*h* ^2^ = 0.15 at age 7 to *h* ^2^ = 0.47 at age 12) on nonverbal ability increased across development. The influence of shared environmental factors decreased across development for nonverbal ability, with the exception at age 9 (*c* ^2^ =  0.23 at age 7, *c* ^2^ = 0.32 at age 9, *c* ^2^ = 0.01 at age 12). The influence of unique environmental factors remained relatively stable (*e* ^2^ = 0.62 at age 7 to *e* ^2^ = 0.53 at age 12) (Table S3).

After accounting for shared genetic and environmental influences in all cross‐lagged paths, a developmental pattern was found whereby age 7 emotional problems predicted lower age 9 nonverbal ability (*E* = 22%) and this subsequently predicted greater emotional problems at age 12 (*E* = 13%). The cross‐lagged path from nonverbal ability to emotional problems was largely explained by shared genetics at age 7 (*A* = 91%) and by shared environment at age 9 (*C* = 61%) (Figure 4b and Table S6). In the reverse direction, lower cognitive ability at age 9 was mostly explained by common environment (*C* = 75%) and by shared genetics at age 12 (*A* = 63%).

### MZ differences design

The cross‐lagged models with MZ difference scores (Table S7 and Supplementary Results: Appendix S1) demonstrated the same pattern of results as our ACE cross‐lagged models, validating the ACE model as a causal inference method.

## Discussion

Emotional problems co‐occur with verbal and nonverbal cognitive deficits. Establishing whether associations reflect causal effects and the likely direction of effects is challenging in observational studies due to unobserved confounding. In this preregistered study, we used cross‐lagged twin models to evaluate whether prospective associations between emotional problems and cognitive ability (and its subdomains; verbal and nonverbal abilities) remain after accounting for genetic and environmental influences shared by twins. Our phenotypic results suggest bidirectional relations between general cognitive ability and emotional problems across ages 7–12. After controlling for effects shared by twins, our results suggest a developmental cascade whereby age 7 emotional problems predict lower nonverbal cognitive ability at age 9, which in turn predicts emotional problems at age 12. No associations were found between nonverbal ability and emotional problems at other time points, nor between verbal ability and emotional problems, which may be due to methodological and sample differences.

Our phenotypic results support findings from observational studies demonstrating that verbal and nonverbal performance have bidirectional relations with symptoms of anxiety or depression across childhood (Flouri et al., 2019; Helland et al., 2018; Romer & Pizzagalli, 2021; Tamayo et al., 2024). However, after controlling for shared genetic and environmental confounders, effects involving nonverbal ability persisted, while associations involving verbal ability did not, mirroring findings at an earlier age (Plomin et al., 2002). The potential causal effect of age 7 emotional problems on age 9 nonverbal ability may indicate that children with emotional problems are not able to take full advantage of educational opportunities due to concentration difficulties or poor school attendance (Finning et al., 2019). This, in turn, may lead to lower memory and reasoning ability (Ritchie, Bates, & Deary, 2015). The effect of age 9 nonverbal ability on age 12 emotional problems could reflect the increasing importance of nonverbal cognition as children transition from primary to secondary school (occurring at age 11 in this sample) (Peng & Kievit, 2020). Furthermore, as children gain greater autonomy, nonverbal ability can help in navigating challenges, such as maintaining peer relationships (Flouri & Panourgia, 2012). This interpretation aligns with competency models of childhood psychopathology whereby achieving a developmental milestone at one stage promotes the development of emerging domains (Masten & Curtis, 2000). It is worth noting that environmental influences accounted for a large proportion of the association from nonverbal ability to emotional problems. For example, families of higher socioeconomic status may have greater resources to invest in their child's emotional development, such as being able to provide a supportive and nurturing home environment (Yeung, Linver, & Brooks‐Gunn, 2002).

In contrast to our hypotheses, we found that none of the cross‐lagged paths between verbal ability and emotional problems remained significant after accounting for confounders. Previous studies that have found a significant effect of language ability on emotional problems measured language skills at ages 3–5, whereas we measured verbal ability at ages 7 and 9 (Bornstein et al., 2013). Language ability may have a larger role in preschool years, when the ability to convey internal mental states helps children self‐regulate (Nelson et al., 2011; Salmon et al., 2016). It may therefore be the case that our results apply only to middle childhood and early adolescence. Our study also differed in the tasks used to measure verbal ability. Other studies tested the ability to read aloud a series of words of increasing difficulty (language expression) (Tamayo et al., 2024), whereas the current study tested the ability to understand the meaning of words (receptive language). Our results suggest that it is unlikely for semantic word knowledge to have a causal effect on emotional problems (and vice versa); however, the causal effect of language expression may still be present, especially at earlier ages. Of note, we observed small phenotypic correlations between verbal performance and emotional problems; therefore, our lack of findings may reflect insufficient power to decompose these paths into genetic and environmental components.

Shared genetics accounted for most of the association from cognitive ability at age 7 (including verbal and nonverbal ability) to age 9 emotional problems, while shared environment additionally accounted for the association from age 9 to 12. In the reverse direction, from emotional problems to cognitive ability, shared environment accounted for associations from age 7 to 9, while genetics explained associations from age 9 to 12. Similar findings were reported by another genetically sensitive study on the influence of general cognitive ability and a psychopathology factor (p‐factor) (von Stumm et al., 2023). The increase in genetic contribution at age 9 may reflect emerging genetic influences or the presence of active gene–environment correlation, such that children with the genetic risk for depression choose to not engage with school, further delaying their cognitive development. These results suggest that the aetiology explaining the co‐occurrence of emotional problems and cognition can vary, depending on the direction of effects.

Strengths of the study include a longitudinal prospective twin dataset, allowing us to control for genetic and environmental confounds. Objective task‐based measures of cognitive ability reduced the possible effect of shared reporter bias. However, our study is not without its limitations. First, the measures for cognitive ability differed across assessment waves. As such, we may be underestimating phenotypic associations between cognition and emotional problems due to measurement differences. However, using different measures is necessary in the assessment of cognitive ability across development. Given that cognition was assessed with valid, age‐appropriate measures, we argue that these are measuring the same underlying construct and are comparable across time. Research finds high stability of general cognitive ability from ages 5 to 12 (Bartels, Rietveld, Van Baal, & Boomsma, 2002). The use of different cognitive measures is also unlikely to bias twin cross‐lagged estimates. In the ACE cross‐lagged model, measurement error is accounted for in the unique environment (E) component. As this time‐ and variable‐specific error does not correlate with other variables, it would not load onto cross‐lagged estimates and therefore should remain unbiased.

Second, data from medical records were not available; therefore, our sample may consist of children who do not meet the clinical threshold for a mood disorder. It could still be possible that cognitive deficits and/or emotional problems may be a causal risk factors in the clinical population. Additionally, the prevalence of depression increases with age and peaks in adolescence; therefore, it remains to be established whether associations between cognition and emotional problems are causal in adolescence (Costello, Mustillo, Erkanli, Keeler, & Angold, 2003). The use of twin designs allows us to test quasi‐causal hypotheses; however, they rely on assumptions including the assumption of random mating (each individual in a population has an equal chance of mating with any other individual, independent of their genetic traits) and equal environments (MZ and DZ co‐twins are exposed to common environmental factors to the same extent). If the equal environmental assumption is not met, there may be an overestimation of genetic effects, whereas a violation of the random mating assumption would underestimate genetic effects. Random mating effects have been observed in cognitive ability and depression and evidence suggests that the equal environment assumption generally holds for parent‐reported psychiatric problems (Kendler, Neale, Kessler, Heath, & Eaves, 1993; Torvik et al., 2022). Where the random mating and equal environments assumptions do not hold, then the estimation of genetics (A) and shared environment (C) may become biased (Falconer's formula). It is noteworthy that nonshared environment (E) would not be directly biased (although, proportionally, E estimates may be influenced owing to the increase in genetic similarity in MZ twins). For the purpose of testing quasi‐causal hypotheses, we do not believe that our conclusions are at risk of bias. Lastly, it remains possible for nonshared confounders to bias our causal estimates. For example, if an illness which only affects one twin simultaneously leads to poor emotional well‐being and cognition, this would create a spurious association between the traits rather than the causal effect of emotional problems on cognition. Nonetheless, quasi‐causal analyses allow for greater certainty in estimating causal associations, as they account for genetic and environmental confounders that have been shown to bias estimates (Baldwin et al., 2023; Grotzinger et al., 2019).

## Conclusions

Quasi‐experimental designs reduce uncertainty in estimating causal associations. Using a longitudinal twin study, we showed that shared genetic and environmental factors explain a large proportion of the variance and covariance between emotional problems and cognitive ability. After controlling for these confounders, we found that emotional problems in young children may be a causal risk factor for nonverbal cognitive deficits, subsequently resulting in higher emotional problems during school age. Our findings suggest that interventions targeting emotional problems in early childhood and nonverbal skills in middle childhood have the potential to improve developmental outcomes.

## Ethical information

TEDS project approval (05.Q0706/228) was granted by the ethics committee for the Institute of Psychiatry, Psychology and Neuroscience at King's College London.

**Key points**

- Emotional problems and cognitive ability co‐occur during childhood.
- It is unclear whether these associations may be causal, and to what extent they may run from cognition to emotion, or vice versa.
- Genetically‐sensitive designs evaluate the extent to which the association between two traits remain after accounting for genetic and environmental confounders.
- Using a twin cross‐lagged model, we examined associations between general cognitive ability, and its sub‐domains of verbal and non‐verbal ability on emotional problems from ages 7 to 12.
- We found that emotional problems at age 7 may be a causal risk factor for lower non‐verbal cognitive ability at age 9 and, subsequently resulting in higher emotional problems at age 12.
- Findings point to the importance of interventions targeting emotional problems in early childhood and non‐verbal cognition in middle childhood.

## Supporting information


**Table S1.** Descriptive statistics.
**Table S2.** Correlation matrix between cognitive ability and emotional problems.
**Table S3.** Proportion of variance in SDQ and G at each age that is accounted for by heritability (h2), shared environment (c2), and nonshared environmental (e2) variance.
**Table S4.** General cognition cross‐Lagged Model and ACE Cross‐lagged Model: Model Fit Indices, Standardized Path Estimates and Percentage of Variance Attributable to Genetic (A), Shared Environmental (C), and Nonshared Environmental (E) Influences.
**Table S5.** Verbal ability Cross‐Lagged Model and ACE Cross‐lagged Model: Model Fit Indices, Standardized Path Estimates and Percentage of Variance Attributable to Genetic (A), Shared Environmental (C), and Nonshared Environmental (E) Influences.
**Table S6.** Nonverbal ability Cross‐Lagged Model and ACE Cross‐lagged Model: Model Fit Indices, Standardized Path Estimates and Percentage of Variance Attributable to Genetic (A), Shared Environmental (C), and Nonshared Environmental (E) Influences.
**Supplementary Methods.** MZ‐differences design to examine the association between cognitive performance and emotional problems.
**Supplementary Results.** MZ‐differences design to examine the association between cognitive performance and emotional problems.
**Table S7.** MZ differences design: phenotypic cross‐lagged paths for the association between general cognition, verbal and non‐verbal abilities and emotional problems.
**Table S8.** Model fit indices for phenotypic and cross‐lagged models.

## Data Availability

The data used in this study come from the Twins Early Development Study (TEDS). Researchers can apply for access to TEDS data for academic purposes through the creation of a Data Access agreement. The full data access policy can be found at the following link: https://www.teds.ac.uk/researchers/teds‐data‐access‐policy.
