# Supplementary material for: Examining the association between cognitive ability and emotional problems across childhood using a genetically informative design: could there be a causal relationship?
Source: J Child Psychol Psychiatry. 2025 Jul 16;66(12):1875–88. doi: 10.1111/jcpp.70008 (PMC12626182; doi:10.1111/jcpp.70008)
Supplement: Supplementary file 1 — Table S1. Descriptive statistics. Table S2. Correlation matrix between cognitive ability and emotional problems. Table S3. Proportion of variance in SDQ and G at each age that is accounted for by heritability (h2), shared environment (c2), and nonshared environmental (e2) variance. Table S4. General cognition cross‐Lagged Model and ACE Cross‐lagged Model: Model Fit Indices, Standardized Path Estimates and Percentage of Variance Attributable to Genetic (A), Shared Environmental (C), and Nonshared Environmental (E) Influences. Table S5. Verbal ability Cross‐Lagged Model and ACE Cross‐lagged Model: Model Fit Indices, Standardized Path Estimates and Percentage of Variance Attributable to Genetic (A), Shared Environmental (C), and Nonshared Environmental (E) Influences. Table S6. Nonverbal ability Cross‐Lagged Model and ACE Cross‐lagged Model: Model Fit Indices, Standardized Path Estimates and Percentage of Variance Attributable to Genetic (A), Shared Environmental (C), and Nonshared Environmental (E) Influences. Supplementary Methods. MZ‐differences design to examine the association between cognitive performance and emotional problems. Supplementary Results. MZ‐differences design to examine the association between cognitive performance and emotional problems. Table S7. MZ differences design: phenotypic cross‐lagged paths for the association between general cognition, verbal and non‐verbal abilities and emotional problems. Table S8. Model fit indices for phenotypic and cross‐lagged models. [file JCPP-66-1875-s001.docx]

**Supporting information**

**Table S1.** Descriptive statistics

**Table S2.** Correlation matrix between cognitive ability and emotional problems

**Table S3.** Proportion of variance in SDQ and G at each age that is accounted for by heritability (h2), shared environment (c2), and nonshared environmental (e2) variance

**Table S4.** General cognition cross-Lagged Model and ACE Cross-lagged Model: Model Fit Indices, Standardized Path Estimates and Percentage of Variance Attributable to Genetic (A), Shared Environmental (C), and Nonshared Environmental (E) Influences

**Table S5.** Verbal ability Cross-Lagged Model and ACE Cross-lagged Model: Model Fit Indices, Standardized Path Estimates and Percentage of Variance Attributable to Genetic (A), Shared Environmental (C), and Nonshared Environmental (E) Influences

**Table S6.** Nonverbal ability Cross-Lagged Model and ACE Cross-lagged Model: Model Fit Indices, Standardized Path Estimates and Percentage of Variance Attributable to Genetic (A), Shared Environmental (C), and Nonshared Environmental (E) Influences

**Supplementary methods.** MZ-differences design to examine the association between cognitive performance and emotional problems

**Supplementary results.** MZ-differences design to examine the association between cognitive performance and emotional problems

**Table S7.** MZ differences design: phenotypic cross-lagged paths for the association between general cognition, verbal and non-verbal abilities and emotional problems

**Table S8**. Model fit indices for phenotypic and cross-lagged models

**Table S1.** Descriptive statistics

|  | N | Mean | SD | Min | Max | Skew | Kurtosis |
| --- | --- | --- | --- | --- | --- | --- | --- |
| 7Y G composite | 5142 | 0 | 1.00 | -3.77 | 4.84 | -0.16 | 0.24 |
| 7Y verbal composite | 5146 | 0 | 1.00 | -3.04 | 5.90 | 0.09 | 0.55 |
| 7Y non-verbal composite | 5176 | 0 | 1.00 | -3.58 | 3.15 | -0.32 | -0.14 |
| 7Y SDQ total (parent-reported) | 7427 | 0 | 1.00 | -1.19 | 4.21 | 1.00 | 0.83 |
| 9Y G composite | 3199 | 0 | 1.00 | -4.03 | 2.11 | -0.53 | -0.20 |
| 9Y verbal composite | 3279 | 0 | 1.00 | -4.58 | 2.50 | -0.26 | -0.15 |
| 9Y non-verbal composite | 3205 | 0 | 1.00 | -3.68 | 1.31 | -0.79 | -0.28 |
| 9Y SDQ total (parent-reported) | 3312 | 0 | 1.00 | -0.92 | 4.43 | 1.31 | 1.52 |
| 12Y G composite | 4213 | 0 | 1.00 | -3.67 | 3.00 | -0.29 | -0.13 |
| 12Y verbal composite | 4412 | 0 | 1.00 | -3.24 | 2.57 | -0.27 | -0.46 |
| 12Y non-verbal composite | 4229 | 0 | 1.00 | -3.93 | 3.05 | -0.36 | 0.28 |
| 12Y SDQ total (parent-reported) | 5691 | 0 | 1.00 | -0.94 | 4.24 | 1.32 | 1.67 |

Note: 1 twin out of each pair was randomly selected, SD= Standard deviation, N=number of participants

**Table S2.** Correlation matrix between cognitive ability and emotional problems

| Variables | 1 | 2 | 3 | 4 | 5 | 6 | 7 | 8 | 9 | 10 | 11 | 12 |
| --- | --- | --- | --- | --- | --- | --- | --- | --- | --- | --- | --- | --- |
| 1.7Y G | 1.00 |  |  |  |  |  |  |  |  |  |  |  |
| 2.7Y verbal | 0.86 | 1.00 |  |  |  |  |  |  |  |  |  |  |
| 3.7Y nonverbal | 0.80 | 0.38 | 1.00 |  |  |  |  |  |  |  |  |  |
| 4.9Y G | 0.41 | 0.38 | 0.30 | 1.00 |  |  |  |  |  |  |  |  |
| 5.9Y verbal | 0.36 | 0.35 | 0.24 | 0.84 | 1.00 |  |  |  |  |  |  |  |
| 6.9Y nonverbal | 0.35 | 0.30 | 0.28 | 0.85 | 0.44 | 1.00 |  |  |  |  |  |  |
| 7.12Y G | 0.47 | 0.44 | 0.34 | 0.57 | 0.46 | 0.50 | 1.00 |  |  |  |  |  |
| 8.12Y verbal | 0.45 | 0.46 | 0.27 | 0.54 | 0.51 | 0.41 | 0.87 | 1.00 |  |  |  |  |
| 9.12Y nonverbal | 0.36 | 0.29 | 0.31 | 0.44 | 0.28 | 0.46 | 0.85 | 0.49 | 1.00 |  |  |  |
| 10.7Y SDQ | -0.04 | -0.06 | -0.01 | -0.13 | -0.08 | -0.13 | -0.08 | -0.08 | -0.05 | 1.00 |  |  |
| 11.9Y SDQ | -0.08 | -0.09 | -0.04 | -0.16 | -0.12 | -0.15 | -0.14 | -0.11 | -0.13 | 0.51 | 1.00 |  |
| 12.12Y SDQ | -0.09 | -0.09 | -0.06 | -0.12 | -0.10 | -0.11 | -0.10 | -0.08 | -0.10 | 0.38 | 0.48 | 1.00 |

Note: 1 twin out of each pair was randomly selected

**Table S3.** Proportion of variance in SDQ and G at each age that is accounted for by heritability (h2), shared environment (c2), and nonshared environmental (e2) variance

|  | h2 | Lower CI | Upper CI | c2 | Lower CI | Upper CI | e2 | Lower CI | Upper CI |
| --- | --- | --- | --- | --- | --- | --- | --- | --- | --- |
| Y7 SDQ | 0.49 | 0.43 | 0.55 | 0.13 | 0.09 | 0.18 | 0.38 | 0.36 | 0.40 |
| Y7 G | 0.38 | 0.33 | 0.43 | 0.30 | 0.26 | 0.35 | 0.32 | 0.30 | 0.33 |
| Y7 verbal | 0.26 | 0.21 | 0.31 | 0.30 | 0.26 | 0.34 | 0.44 | 0.42 | 0.46 |
| Y7 non-verbal | 0.15 | 0.12 | 0.17 | 0.23 | 0.21 | 0.26 | 0.62 | 0.60 | 0.64 |
| Y9 SDQ | 0.19 | 0.07 | 0.28 | 0.34 | 0.27 | 0.42 | 0.47 | 0.43 | 0.52 |
| Y9 G | 0.20 | 0.15 | 0.27 | 0.49 | 0.43 | 0.53 | 0.31 | 0.29 | 0.33 |
| Y9 verbal | 0.12 | 0.08 | 0.17 | 0.45 | 0.41 | 0.48 | 0.43 | 0.40 | 0.45 |
| Y9 non-verbal | 0.29 | 0.23 | 0.35 | 0.32 | 0.27 | 0.37 | 0.39 | 0.36 | 0.42 |
| Y12 SDQ | 0.55 | 0.51 | 0.60 | 0.02 | 0.00 | 0.05 | 0.43 | 0.36 | 0.47 |
| Y12 G | 0.47 | 0.41 | 0.53 | 0.11 | 0.06 | 0.16 | 0.42 | 0.40 | 0.46 |
| Y12 verbal | 0.42 | 0.36 | 0.47 | 0.04 | 0.00 | 0.08 | 0.54 | 0.52 | 0.57 |
| Y12 non-verbal | 0.47 | 0.39 | 0.52 | 0.01 | 0.00 | 0.02 | 0.53 | 0.48 | 0.58 |

Note: h2 = Additive genetics, C2 = Shared environment, e2 = Unique environment. CI = 95% Confidence Intervals. SDQ = Strength and Difficulties Questionnaire; G= General cognitive ability

**Table S4.** General cognition Cross-Lagged Model and ACE Cross-lagged Model: Model Fit Indices, Standardized Path Estimates and Percentage of Variance Attributable to Genetic (A), Shared Environmental (C), and Nonshared Environmental (E) Influences

|  | A | C | E | A (%) | C(%) | E (%) |
| --- | --- | --- | --- | --- | --- | --- |
| Stability paths |  |  |  |  |  |  |
| 7Y g-composite 🡪 9Y g-composite | 0.92  (0.85, 0.98) | 0.28  (0.17, 0.36) | 0.00  (0.00, 0.02) | 72%  (62, 80)% | 28%  (19, 3)% | 0% (0, 2)% |
| 9Y g-composite 🡪 12Y g-composite | 0.99  (0.98, 1.00) | 0.60  (0.46, 0.70) | 0.06  (0.00, 0.11) | 68% (59, 77)% | 30%  (21, 38)% | 2% (0, 5)% |
| 7Y SDQ🡪 9Y SDQ | 0.78  (0.72, 0.85) | 0.42  (0.14, 0.63) | 0.37  (0.32, 0.41) | 57%  (39, 70)% | 15%  (5, 28)% | 27% (24, 33)% |
| 9Y SDQ🡪 12Y SDQ | 0.87  (0.80, 0.93) | 0.39  (0.00, 0.89) | 0.35  (0.30, 0.41) | 68%  (48, 77)% | 5%  (0, 20)% | 26% (22, 32)% |
| Correlational paths |  |  |  |  |  |  |
| 7Y g-composite ↔ 7Y SDQ | -0.10  (-0.14, -0.05) | -0.21  (0.30, 0.10) | -0.01  (-0.04, 0.02) | 49%  (29, 71)% | 47%  (27, 66)% | 4% (0, 12)% |
| 9Y g-composite ↔ 9Y SDQ | 0.00  (-0.05, 0.00) | -0.13  (-0.27, 0.04) | -0.02 (-0.05, 0.01) | 28% (6, 58)% | 67%  (39, 89)% | 5% (0, 15)% |
| 12Y g-composite ↔ 12Y SDQ | 0.05  (0.00, 0.11) | -0.11  (-0.54, 0.21) | -0.04 (-0.08, -0.01) | 1%  (0, 1)% | 40%  (3, 74)% | 59% 38, 94)% |
| Cross-lagged paths |  |  |  |  |  |  |
| 7Y g-composite 🡪 9Y SDQ | -0.15  (-0.21, -0.07) | -0.03  (-0.20, 0.00) | -0.02 (-0.05, 0.00) | 61% (33, 87)% | 34%  (7, 70%) | 6%  (0, 14)% |
| 9Y g-composite 🡪 12Y SDQ | 0.00  (-0.18, 0.00) | -0.72  (-1.00, -0.30) | -0.04  (-0.08, 0.00) | 9% (0, 29)% | 79%  (53, 96)% | 12% (1, 23)% |
| 7Y SDQ 🡪 9Y g-composite | 0.00  (-0.03, 0.00) | -0.31  (-0.46, -0.21) | -0.07  (-0.10, -0.03) | 7% (0, 21)% | 73%  (56, 85)% | 19% (10, 29) % |
| 9Y SDQ 🡪 12Y g-composite | 0.00  (-0.06, 0.00) | -0.09  (-0.26, 0.00) | -0.04  (-0.08, 0.00) | 22% (1, 77)% | 57%  (9, 89)% | 21% (3, 58)% |

*Note.* All estimates were obtained after regressing for age and sex; numbers in parentheses are 95% confidence intervals; -2LL =negative 2 times log likelihood. *df =* degrees of freedom; AIC= Akaike information criterion; CFI: Bentler comparative fit index; RM SEA= root mean square error of approximation; SDQ = Strength and Difficulties Questionnaire. Missing values indicate paths that were dropped for model convergence.

**Table S5.** Verbal ability Cross-Lagged Model and ACE Cross-lagged Model: Model Fit Indices, Standardized Path Estimates and Percentage of Variance Attributable to Genetic (A), Shared Environmental (C), and Nonshared Environmental (E) Influences

| Path | A | C | E | A (%) | C(%) | E (%) |
| --- | --- | --- | --- | --- | --- | --- |
| Stability paths |  |  |  |  |  |  |
| 7Y verbal 🡪 9Y verbal | 0.92  (0.82, 1.00) | 0.00  (0.00, 0.14) | 0.00 (0.00, 0.02) | 96%  (87,100)% | 4%  (0, 17)% | 0%  (0, 3)% |
| 9Y verbal 🡪 12Y verbal | 0.99  (0.97, 1.00) | 0.51 (0.26, 0.77) | 0.02 (0.00, 0.07) | 73%  (59, 88)% | 24%  (10, 36)% | 3%  (0,9)% |
| 7Y SDQ🡪 9Y SDQ | 0.79  (0.73, 0.85) | 0.46  (0.21, 0.64) | 0.37 (0.32, 0.42) | 55%  (37, 69)% | 17%  (6, 29)% | 28%  (23, 34)% |
| 9Y SDQ🡪 12Y SDQ | 0.87  (0.80, 0.93) | 0.76 (0.04, 1.00) | 0.36 (0.31, 0.42) | 64%  (46, 78)% | 10%  (0, 23)% | 26% (22,32)% |
| Correlational paths |  |  |  |  |  |  |
| 7Y verbal ↔ 7Y SDQ | -0.09  (-0.13, -0.05) | -0.22 (-0.30, -0.12) | 0.02  (-0.01, 0.05) | 38%  (23, 58)% | 52%  (31, 73)% | 10%  (0, 20)% |
| 9Y verbal ↔ 9Y SDQ | 0.00  (-0.12, 0.00) | -0.04 (-0.09, 0.02) | -0.01 (-0.04, 0.02) | 42%  (18, 78)% | 48%  (2, 77)% | 10%  (0, 29)% |
| 12Y verbal ↔ 12Y SDQ | 0.06  (0.00, 0.77) | 0.13 (-0.52, 0.78) | -0.01 (-0.04, 0.03) | 28%  (0, 47)% | 44%  (20, 92% | 27%  (4, 75)% |
| Cross-lagged paths |  |  |  |  |  |  |
| 7Y verbal 🡪 9Y SDQ | -0.12  (-0.18, -0.08) | - | 0.00  (-0.03, 0.00) | 46%  (18, 78)% | 35%  (8, 59)% | 19%  (9, 28)% |
| 9Y verbal 🡪 12Y SDQ | 0.00  (-0.12, 0.00) | -0.23 (-0.91, -0.01) | 0.00 (-0.04, 0.00) | 40%  (6, 83)% | 42%  (1, 78)% | 18%  (9, 31)% |
| 7Y SDQ 🡪 9Y verbal | 0.00  (-0.03, 0.00) | -0.12  (-0.24, -0.03) | -0.01 (-0.04, 0.00) | 26%  (3, 90)% | 68%  (21, 98)% | 6%  (0, 41%) |
| 9Y SDQ 🡪 12Y verbal | 0.00  (0.00, 0.00) | -0.01  (-0.24, 0.00) | 0.00 (-0.06, 0.00) | 94%  (63, 100)% | 5%  (0, 47)% | 1%  (0, 12)% |

*Note.* All estimates were obtained after regressing for age and sex; numbers in parentheses are 95% confidence intervals; -2LL =negative 2 times log likelihood. *df =* degrees of freedom; AIC= Akaike information criterion; CFI: Bentler comparative fit index; RMSEA= root mean square error of approximation; SDQ = Strength and Difficulties Questionnaire. Missing values indicate paths that were dropped for model convergence.

**Table S6.** Nonverbal ability Cross-Lagged Model and ACE Cross-lagged Model: Model Fit Indices, Standardized Path Estimates and Percentage of Variance Attributable to Genetic (A), Shared Environmental (C), and Nonshared Environmental (E) Influences

| Path | A | C | E | A (%) | C(%) | E (%) |
| --- | --- | --- | --- | --- | --- | --- |
| Stability paths |  |  |  |  |  |  |
| 7Y nonverbal 🡪 9Y nonverbal | 0.71 (0.59, 0.81) | - | 0.00 (0.00, 0.00) | 98%  (97, 99)% | 1%  (1, 2)% | 0% (0, 1)% |
| 9Y nonverbal 🡪 12Y nonverbal | 0.77 (0.68, 0.85) | 0.52 (0.02, 1.00) | 0.08 (0.03, 0.14) | 79% (64, 94)% | 12% (0, 23)% | 9% (3, 15)% |
| 7Y SDQ🡪 9Y SDQ | 0.82 (0.76, 0.87) | 0.22  (0.06, 0.35) | 0.34 (0.31, 0.39) | 69%  (64, 74)% | 6% (2, 10)% | 25% (22, 28)% |
| 9Y SDQ🡪 12Y SDQ | 0.83  (0.79, 0.86) | - | 0.35 (0.31, 0.40) | 74% (69, 78)% | 2%  (0, 4)% | 24% (21, 29)% |
| Correlational paths |  |  |  |  |  |  |
| 7Y nonverbal ↔ 7Y SDQ | -0.02 (-0.09, 0.00) | - | -0.03 (-0.06, 0.00) | 28% (0, 89)% | 0 %  (0, 0)% | 72% (11, 100)% |
| 9Y nonverbal ↔ 9Y SDQ | 0.06 (0.00, 0.17) | -0.23 (-0.33, -0.11) | -0.02 (-0.05, 0.02) | 25%  (3, 64)% | 72% (42, 96)% | 3% (0, 10)% |
| 12Y nonverbal ↔ 12Y SDQ | 0.00 (0.00, 0.00) | 0.52 (0.00, 0.95) | -0.03  (-0.08, 0.01) | 46%  (13, 79)% | 27% (1, 54)% | 26%  (2, 70)% |
| Cross-lagged paths |  |  |  |  |  |  |
| 7Y nonverbal 🡪 9Y SDQ | -0.17 (-0.25, -0.11) | - | 0.00 (-0.04, 0.00) | 91% (74, 100)% | 2%  (1, 4)% | 7%  (0, 23)% |
| 9Y nonverbal 🡪 12Y SDQ | 0.00 (0.00, 0.00) | -0.79 (-1.00, -0.42) | -0.07 (-0.10, -0.02) | 26%  (5, 37)% | 61% (45, 83)% | 13% (1, 32)% |
| 7Y SDQ 🡪 9Y nonverbal | 0.00 (0.00, 0.00) | -0.45 (-0.60, -0.32) | -0.07 (-0.10, -0.02) | 3%  (0, 14)% | 75% (63, 92)% | 22% (7, 33)% |
| 9Y SDQ 🡪 12Y nonverbal | -0.12 (-0.19, -0.03) | - | -0.04 (-0.09, 0.00) | 63% (21, 97)% | 11%  (0, 30)% | 26%  (1, 63)% |

*Note.* All estimates were obtained after regressing for age and sex; numbers in parentheses are 95% confidence intervals; -2LL =negative 2 times log likelihood. *df =* degrees of freedom; AIC= Akaike information criterion; CFI: Bentler comparative fit index; RMSEA= root mean square error of approximation; SDQ = Strength and Difficulties Questionnaire. Missing values indicate paths that were dropped for model convergence.

**Supplementary methods.** MZ-differences design to examine the association between cognitive performance and emotional problems

In an MZ twin differences design, twin differences in one trait or exposure are used to predict differences in another trait or outcome. MZ twins share 100% of their genes and environment, so shared genes and shared environment cannot explain differences between two individuals in an MZ pair (Supplementary methods). Thus, asking if MZ twin differences on one trait predicts the same in another is akin to asking if the relationship between the two traits persists after controlling for all genetic and shared environmental factors common to the two traits. If the association does not persist, it can be concluded that it is attributable to overlapping genetic and shared environmental factors and is thus not causal. If the relationship is a causal one, then it should remain after accounting for common genetic and environmental factors. This is because all traits are under the influence of nonshared environmental influences (the third law of behavioural genetics)^1^. Nonshared environmental influences are all those influences that make MZ twins different to one another. So if one trait causes another, the nonshared environmental influences on the causal trait should explain some of the covariance between them. Thus, if an association between two traits remains after accounting for common genetic and shared environmental factors then it *could* be causal (it could also be attributable to nonshared environmental confounding). An MZ twin differences design is one way of controlling for common genetic and shared environmental factors, but this can also be achieved using a sample of both MZ and DZ twins and applying biometric modelling to evaluate whether the non-shared environmental influences on one trait predict another. If so, the association may be causal. In this paper we apply biometric models to a sample of MZ and DZ twins because this has more power than focussing only on MZ twins. However, in the supplementary materials we include an MZ difference cross-lagged model for comparison. This model asks the same question we do in our main analyses, using only the MZ twins (it thus has less power than the model we present).

References

1. Turkheimer, E. (2000). Three laws of behavior genetics and what they mean. *Current directions in psychological science*, *9*(5), 160-164

**Supplementary results.** MZ-differences design to examine the association between cognitive performance and emotional problems

*Cross-lagged twin model using a MZ differences design*

The results of the cross-lagged models with MZ differences scores (Table S7) demonstrated the same pattern of results from as our ACE twin cross-lagged model. In the general cognitive performance MZ differences model, general cognitive performance showed a potential causal path from age 7 cognitive performance to age 9 emotional problems. When general cognitive performance was split into its subdomains, only non-verbal ability demonstrated significant cross-lagged paths. Mirroring results obtained from the ACE cross-lagged model, it was found that higher age 7 emotional problems prospectively reduced non-verbal ability at age 9), this then led to an increase in age 12 emotional problems. Therefore, validating the ACE model as a possible causal inference method.

**Table S7.** MZ differences design: phenotypic cross-lagged paths for the association between general cognition, verbal and non-verbal abilities and emotional problems

| General cognition | | Verbal | | Non-verbal | |
| --- | --- | --- | --- | --- | --- |
| Path | Phenotypic | Path | Phenotypic | Path | Phenotypic |
| Stability paths |  |  |  |  |  |
| 7Y G🡪 9Y G | 0.04  (0.00, 0.09) | 7Y verbal 🡪 9Y verbal | 0.03 (-0.02, 0.07) | 7Y nonverbal 🡪 9Y nonverbal | -0.03 (-0.07, 0.01) |
| 9Y G🡪 12Y G | 0.10  (0.04, 0.16) | 9Y verbal 🡪 12Y verbal | 0.01 (-0.05, 0.06) | 9Y nonverbal 🡪 12Y nonverbal | 0.13 (0.07, 0.18) |
| 7Y SDQ🡪 9Y SDQ | 0.42  (0.38, 0.46) | 7Y SDQ🡪 9Y SDQ | 0.38 (0.33, 0.43) | 7Y SDQ🡪 9Y SDQ | 0.38 (0.33, 0.42) |
| 9Y SDQ🡪 12Y SDQ | 0.38  (0.33, 0.42) | 9Y SDQ🡪 12Y SDQ | 0.42 (0.38, 0.46) | 9Y SDQ🡪 12Y SDQ | 0.42 (0.38, 0.46) |
| Correlational paths |  |  |  |  |  |
| 7Y G ↔ 7Y SDQ | 0.00  (-0.03, 0.03) | 7Y verbal ↔ 7Y SDQ | 0.03 (-0.01, 0.07) | 7Y nonverbal ↔ 7Y SDQ | -0.03 (-0.06, 0.01) |
| 9Y G ↔ 9Y SDQ | -0.06  (-0.11, -0.02) | 9Y verbal ↔ 9Y SDQ | 0.00 (-0.04, 0.05) | 9Y nonverbal ↔ 9Y SDQ | -0.06 (-0.10, -0.01, |
| 12Y G ↔ 12Y SDQ | -0.06  (-0.10, -0.02) | 12Y verbal ↔ 12Y SDQ | 0.00 (-0.04, 0.04) | 12Y nonverbal ↔ 12Y SDQ | -0.09 (-0.15, -0.03) |
| Cross-lagged paths |  |  |  |  |  |
| 7Y SDQ🡪 9Y G | -0.05 (-0.09, -0.01) | 7Y SDQ🡪 9Y verbal | 0.02 (-0.03, 0.06) | 7Y SDQ🡪 9Y nonverbal | -0.07 (-0.11, -0.02) |
| 9Y SDQ 🡪 12Y G | -0.03 (-0.07, 0.02) | 9Y SDQ 🡪 12Y verbal | 0.02 (-0.03, 0.06) | 9Y SDQ 🡪 12Y nonverbal | -0.05 (-0.10, 0.00) |
| 7Y G 🡪 9Y SDQ | 0.00 (-0.04, 0.04) | 7Y verbal 🡪 9Y SDQ | 0.00 (-0.04, 0.04) | 7Y nonverbal 🡪 9Y SDQ | 0.00 (-0.04, 0.04) |
| 9Y G🡪 12Y SDQ | -0.04 (-0.09, 0.00) | 9Y verbal🡪 12Y SDQ | 0.01 (-0.04, 0.06) | 9Y nonverbal🡪 12Y SDQ | -0.05 (-0.09, -0.01) |
| Phenotypic cross-lagged model fit | AIC= 65584.24  CFI=0.94  TFI= 0.79  RMSEA=0.04 | AIC= 67662.903  CFI=0.94  TFI= 0.79  RMSEA=0.07 |  | AIC= 64726.933  CFI=0.95  TFI= 0.81  RMSEA=0.04 |  |

Note: Difference score was calculated by subtracting Twin 2 scores from Twin 1.

**Table S8**. Model fit indices for phenotypic and cross-lagged models

| *Phenotypic and ACE cross-lagged model fit against saturated model* | *AIC* | *CFI/TFI* | *RMSEA* | *diffLL(df)* | *p* |
| --- | --- | --- | --- | --- | --- |
| General cognitive performance on emotional problems | 30984.2 | 0.99/0.99 | 0.02 | 146714.2(57865) | <0.05 |
| Verbal ability on emotional problems | 29585.7 | 0.99/0.98 | 0.02 | 145315.7 (57865) | <0.05 |
| Non-verbal ability on emotional problems | 29596.9 | 0.99/0.98 | 0.02 | 135615.8 (53252) | <0.05 |

*Note:* AIC= Akaike information criterion; CFI: Bentler comparative fit index; RMSEA= root mean square error of approximation; SDQ= strength and difficulties questionnaire.
